# More knowledge, more choices? How peer recognition of physicians’ knowledge sharing affect patients’ consultation in online health communities

**DOI:** 10.3389/fpubh.2024.1376887

**Published:** 2024-10-18

**Authors:** Zhen Xu, Xiaochen Liu, Lingguang Meng, Xuanxuan Lyu

**Affiliations:** ^1^School of Communication, East China University of Political Science and Law, Shanghai, China; ^2^School of Economics and Management, Liaoning University of Technology, Jinzhou, China; ^3^School of Journalism and Communication, Shanghai University, Shanghai, China; ^4^International Relations Department, Beihang University, Beijing, China

**Keywords:** online health communications, patient consultation, knowledge sharing, peer recognition, telemedicine

## Abstract

**Background:**

The advent of telemedicine has revolutionized healthcare consultations, primarily due to the digital era and global health concerns. Online healthcare communities (OHCs) have emerged as platforms for physicians to share health-related articles, promoting digital public health awareness and knowledge dissemination. The continuous dissemination of health knowledge by physicians online is considered a crucial driving force in attracting patients to seek online consultations.

**Methods:**

Based on the elaboration likelihood model and the information overload theory, this study explores how persuasive messages from other patients’ peer recognition, including knowledge popularity and attractiveness, affect patients’ consultation decisions. Additionally, the study examines the three-way interaction between knowledge popularity, attractiveness, and quantity in shaping patient consultations. Using data collected from 2,676 physicians on haodf.com, this study established an ordinary least squares (OLS) regression model with time and city fixed effects to test the hypothesis.

**Results:**

The results show that: (1) peer recognition (knowledge popularity and attractiveness) from other patients positively impacts patients’ consultation; (2) knowledge attractiveness positively moderate the relationship between knowledge popularity and patients’ consultation; (3) there is a three-way effect of knowledge popularity, knowledge attractiveness, and knowledge quantity on patients’ consultation.

**Conclusion:**

Our findings offer valuable guidance for platform design and healthcare practitioners, boosting patient-physician engagement in online healthcare communities.

## 1 Introduction

The use of telemedicine has completely changed the way in which traditional health consultations are conducted (1). As a result of the digital era, patients consult with physicians in a different way, as well as the fact that healthcare concerns prevail throughout the world (2). More patients choose to seek medical treatment in online health communities (OHCs) (3). In OHCs, physicians can share articles related to health knowledge and issues, increasing patient awareness of health and assisting government and related institutions in spreading professional knowledge concerning health and prevention (4). Ideally, OHC platforms should be sustainable to maintain physicians’ motivation to share their knowledge (5, 6). Physicians’ continuous production of free or paid knowledge-sharing content not only aids the dissemination of medical knowledge but also supports patients in making informed medical decisions. This, in turn, attracts more patient engagement and consultation activities on the platform (7). Patient engagement and the act of seeking advice are essential for the ongoing sustainability of the platform. Hence, it is highly important to investigate the mechanisms that influence patient consultation behavior in OHC.

Knowledge sharing in the context of OHCs refers to content produced by physicians (e.g., articles). This kind of content sharing has been considered to be a critical participative behavior in online communities (8). User participation of online communities is regarded as an integrated concept, typically measured by the number of postings, replies, views, and reading time (9). Regarding OHC, health care professionals along with patients make up two major groups of an information-sharing community. Health care professionals contribute by creating health-content in the form of medical knowledge (10), while patients engage in the community by sharing their experiential knowledge and reflections in the form of comments made on their own consultations (11). In OHC, doctors providing health-information may encourage the patient’s engagement (7). Peer recognition might support content creation (12). Peer recognition represents that the effort of content generator has been appreciated by other users in the same community (13). Generally, peer recognition is considered a positive form of feedback that encourages and rewards the work of content generators (12). Similarly, in the context of OHCs, peer recognition refers to the acknowledgment by other patients of a physician’s knowledge sharing. This recognition, such as the number of articles read and the total number of followers, plays a significant role in influencing new patients’ decisions when selecting a physician for consultation. Peer recognition can be reflected by persuasive messages (including popularity and attractiveness) from other patients, which may indicate the extent to which patients acknowledge the knowledge sharing of the physician. However, the impact of peer recognition from other patients on patient consultation is still unclear.

Besides, not only persuasive messages of other patients, the amount of knowledge sharing has always been seen as a controversial variable. Quantity of online consumer reviews has been shown to positively influence consumer purchase intentions, and consumer purchasing intentions increase as the number of reviews increases (14). Further, when the quantity of information (e.g., reviews) increases, based on the theory of information overload, consumers may find decision-making more difficult due to the excessive amount of information. In such cases, they might rely on other signals, such as an increase in popularity, to make their decisions (15). In OHCs, the mechanism by which the quantity of a physician’s knowledge sharing interacts with peer recognition cues from other patients (such as knowledge popularity and knowledge attractiveness) to influence patient consultation behavior remains unclear. Hence, it is crucial to consider the three-way relationship involving knowledge popularity, knowledge attractiveness, and knowledge quantity as peripheral cues and their joint effects on patient consultations.

Although previous literature has explored aspects of how free knowledge sharing in online health communities can help physicians gain popularity, attract more patients, and enhance physician-patient relationships and trust through the quality and quantity of shared content (16), the interaction between knowledge popularity, knowledge attractiveness, and knowledge quantity remains unexplored. The elaboration likelihood model (ELM) suggests that decision-making is typically influenced by a combination of various cues. However, existing research lacks sufficient exploration of the interaction effects among multiple cues on user decision-making. To address these gaps, this study seeks to explore the influence of peer recognition from other patients on patient consultation selection intention through a three-way interaction model. To this end, the present study draws upon the framework of the ELM to elucidate how peripheral cues (e.g., knowledge popularity and knowledge attractiveness) that are formulated by other patients who appreciate physicians’ knowledge sharing behavior might influence patient consultations within OHCs. This study analyzed a three-way interaction model to explore how knowledge popularity, knowledge attractiveness, and knowledge quantity might impact patient consultations. This study developed hypotheses using a primary data of 2,676 physicians fetched from haodf.com, during the period of April 2022 and September 2022. The research helps to understand the interactive effects of multiple cues in physician knowledge sharing within online health communities and offers practical insights for platforms and physicians in sustaining patient engagement and consultation behavior.

## 2 Theoretical background and hypotheses development

### 2.1 Elaboration likelihood model

According to the Elaboration Likelihood Model (ELM), the effectiveness of persuasive messages depends on the likelihood that the receiver will actually process the content of the message thoughtfully (17). The ELM theory is commonly used in fields such as marketing and sociology to better understand how people receive and digest information to make decisions (18). The central route involves careful and thoughtful consideration of the arguments presented, while the peripheral route relies on superficial cues such as the attractiveness or credibility of the source. Insights from applying ELM in advertising reveal that persuasive message content can significantly influence an individual’s attitude toward adopting a new information system (19). The central route emphasizes deep and thoughtful processing of information, focusing on the quality and strength of the arguments presented (20). This approach requires substantial cognitive effort and is used when individuals are motivated and capable of thoroughly evaluating the message content. Conversely, the peripheral route depends on superficial cues rather than the argument quality (21). In ELM, source attractiveness often serves as a peripheral cue, whereas attributes like argument strength, informational quality, logic, and reasoning are considered central route features. Petty and Cacioppo (22) clearly describe attractiveness as a superficial peripheral cue. The peripheral route requires much less cognitive effort and is used when people are not motivated to, or not able to, process the information deeply. Since peripheral cues require less cognitive effort than central cues, they more easily stimulate consumer decisions. In online health communities (OHCs), other patients’ recognition of a physician’s knowledge sharing, such as the quantity, popularity, and attractiveness of the shared knowledge, are all peripheral cues. However, how these peripheral cues collectively influence patients’ consultation decisions is not yet clear. Based on ELM, this study aims to explore how the interactions among the three peripheral cues—knowledge quantity, knowledge popularity, and knowledge attractiveness—affect patient consultation behavior.

### 2.2 Information overloading theory

Due to the widespread use of computers and the internet, people are faced with an abundance of information online that is far beyond their ability to manage and adapt to (23). This situation is referred to as information overload (24). The decision-making process may be affected when people are faced with and struggle to cope with a tremendous amount of information when they have a finite capacity to handle messages (25). Generally, information overload occurs when the requirements for handling information exceed one’s capacity to handle the information within the time available (26). Information overload normally leads to feelings of being overwhelmed, confusion, or even psychosocial pressure (25). When people are constantly dealing with multiple sources of information, they will experience mental exhaustion. As a result of excessive information overload, users will be exhausted and stressed, as processing excessive information will drain their mental resources, energy, and enthusiasm, leading to fatigue (27). There should be a greater prevalence of psychological ill-being and corresponding traits among individuals who have recently been drowning in a sea of information from search engines that provide healthcare information (28). Overall, when people face an abundance of information, their decision-making may be affected.

### 2.3 Knowledge sharing in OHCs

Knowledge sharing pertains to the process by which an individual imparts knowledge to fellow members within a specified community (29). Knowledge sharing is one of the most challenging aspects of online Q&A communities (30). There has been research done on the antecedents of knowledge sharing in online communities, which include motivational factors such as intrinsic motivators, altruistic motivation, pleasure, accomplishment, and self-efficacy (31–33), technical factors including perceived usefulness, and perceived ease of use (34, 35), social factors including community trust, reciprocity norms, and community incentives (36, 37). However, there has been relatively little research conducted on knowledge sharing of health professionals in online health communities (38). There are two distinct types of knowledge contributors: health professionals and non-health professionals (38). In this study, health professionals were adopted as knowledge contributors. Considering the limited research on peer recognition messages resulting from knowledge sharing, this study will examine their influence on patient consultation behavior. Specifically, the paper aims to investigate how peripheral cues (e.g., knowledge popularity and knowledge attractiveness) that are formulated by other patients impact patient consultation behavior.

### 2.4 Peer recognition and patient consultation

Peer recognition, also known as social recognition, is defined as an attitude or practice by which a certain quality of a social group or individual is affirmed (39). In online communities, peer recognition is a crucial mechanism that influences user engagement and community sustainability (40). Peer recognition in online content contribution refers to administering acknowledgment and reward mechanisms to contributors based on their reputation and feedback in the community. This form of recognition impacts the volume and quality of content generated since contributors are motivated to contribute content of better quality to receive positive feedback and recognition from their peers (41). Similarly, in online product communities, peer recognition pertains to other users’ acknowledgment of a user’s contributions, which in turn consolidates the user’s position and encourages the users to engage actively in the community. This recognition helps to build trust within the community leading to enhance user engagement and continuous community interactions (42). Also, social recognition and psychological ownership significantly impact intentions to share content in online communities, as they are motivators for content sharing and community engagement, but also they provide a sense of belonging and value among community members (43).

Peer recognition is important in online communities because it provides feedback on the value of individual contributions, reinforces the trustworthiness of the individual, and further encourages a user’s active engagement and quality content production. It also provides feedback regarding the worth of contributions, increases the trustworthiness of the individual, encourages active participation, and assists in sustaining the community by providing engagement and motivation for members to engage and provide high quality content. In OHCs, this study refer to peer recognition as the acknowledgment by other patients of a physician or source’s knowledge sharing, which can significantly impact the decision-making process of patients when selecting a physician to consult. This study will explore how peer recognition cues (knowledge popularity and knowledge attractiveness) from other patients impact patient consultation behavior.

Knowledge popularity in online content communities can refer to the degree to which discrete knowledge or forms of content are widely accessed, utilized, and valued in the community (44). The measurement of knowledge popularity usually include the number of views, shares, comments, and likes. Beyond popularity, Le considers the role of word of mouth (WOM) types and content characteristics on online engagement using views, replies, likes, comments, and share as metrics of popularity (45). Similarly, Uddin highlight the use of the metrics of shares, likes, and comments to predict popularity in online news (46). In the context of CQA services, Liu et al. focus on question popularity through views and comments, suggesting the use of these metrics to consider user interest and engagement (44). The total number of views has also been widely used to predict the popularity of online news articles (47). A similar analysis will be expanded to the OHC context, where the number of articles read would be used as a dynamic proxy of how popular the knowledge shared by physicians in the articles is. Therefore, knowledge popularity could have implications on patient consultation behavior in OHCs context. As a result, the following hypotheses is posited:

*H*1: Knowledge popularity is positively related to patients’ consultation.

Knowledge attractiveness in online content communities is typically defined by metrics such as the number of followers, likes, shares, and comments, which indicate the level of engagement and interest from community members (48). This attractiveness is crucial for understanding user behavior and content popularity. For instance, in paid Q&A platforms, payment attractiveness is gaged by factors like the number of likes, followers, public answers, and reviews that a knowledge contributor receives, indicating how likely users are to engage with and pay for the knowledge provided (48). Similarly, in knowledge-sharing platforms, knowledge attractiveness is measured by the number of followers and the engagement with the content, including likes and shares. This reflects how recommendation algorithms can influence user interests and attract followers (49). Furthermore, the attractiveness of content on social media platforms is measured by the number of followers, the conversion rate of followers, and overall engagement metrics such as likes and comments (50).

In the Instagram context, the ratio of followers positively affect the perceived influencer credibility, the influencer credibility positively affects the purchase intention indirectly (51). Since the number of followers represents that the interests from people in this account and the information generated by this account (52). People tends to identify the attractiveness of an account based on the number of followers (53). Thus, the user with more followers has been considered more attractive (54). Physicians on OHC platforms share knowledge to other users (patients and other physicians) (38). Thus, when other users find the knowledge shared by the physician is attractive, they may follow this physician. Physicians with high follower ratio means that the knowledge they shared is more attractive than others. Therefore, the following hypotheses is proposed:

*H*2: Knowledge attractiveness is positively related to patients’ consultation.

In the Instagram context, when the follower number is high, the account is considered more popular and likable (52). In the OHCs context, when a physician with high follower ratio, the articles published by this physician might be read by these followers, and other users may think this physician is credible and famous, then other patients may trust in physician with more followers. Patients tend to trust in famous physicians, since they believe that a famous physician is more professional and with more experience. And a physician with more followers may means that this physician has been recognized by other patients. According to the herd effect, consumers tend to make consumption decisions based on popularity cues (55). Thus, people may consult in this physician. In situations with high knowledge attractiveness (the conversion rate of followers), knowledge popularity (number of articles read) will have a greater impact on patient consultations.

Therefore, the following hypotheses is proposed:

*H*3: Knowledge attractiveness positively moderate the relationship between knowledge popularity and patients’ consultation.

### 2.5 Moderators of knowledge quantity

The Elaboration Likelihood Model (ELM) is a key model for understanding how persuasive messages influence attitudes (56). According to ELM, people can change their attitudes through two different routes: the central route or the peripheral route. The central route involves careful and thoughtful consideration of the message, which happens when the message is personally relevant and individuals are motivated to process it deeply (19). The more effort and thought people put into processing the message, the more likely they are to be influenced by its actual content and quality (central cues). In contrast, the peripheral route is a route which requires less cognitive effort, and focuses on the superficial aspects of the message, such as who the source is, or how many times you have heard the message (57). In general, when the motivation is relatively low to process the message systematically, people are more likely to be influenced by peripheral cues. In addition, peripheral cues are typically more noticeable and have a more immediate impact on our decisions than central cues. Peripheral cues often are more effective than central cues in affecting people decisions, particularly when they are less motivated to engage in extensive message processing (56).

**Figure 1:**
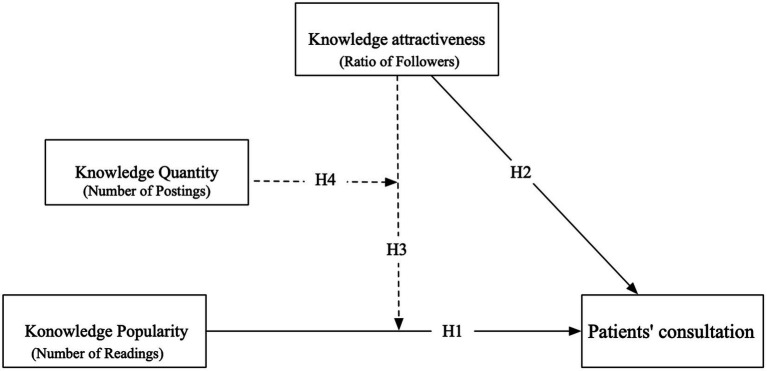
Research model.

This research conceptualized knowledge attractiveness, knowledge quantity, and knowledge popularity as peripheral route cues. Knowledge quantity, being the number of articles a physician has shared, is more easily recognized by patients. Based on Information Overloading Theory, it has been proposed that when consumers are faced with an abundance of articles, they tend to trust their peers’ decisions (23). Therefore, when a physician has a high quantity of shared knowledge, peer recognition cues from other patients play a more significant role in consumer decision-making. As a result, the positive moderating effect of knowledge attractiveness is strengthened. Consequently, we propose a hypothesis of a three-way interaction among these factors (Figure 1).

Therefore, the following hypotheses are proposed:

*H*4: There is a three-way effect of knowledge popularity, knowledge attractiveness, and knowledge quantity on patients’ consultation. The positive moderating effect of knowledge attractiveness is strengthened when knowledge quantity is high.

## 3 Methodology

### 3.1 Data and description of variable

The study was conducted within the context of the “Good Doctor Online” website,^1^ one of China’s most reputable healthcare websites. On this website, there are over 8,847 hospitals and over 545,586 physicians from hospitals registered with the site. This website is increasingly being used by patients to locate a physician who is appropriate for consultations. A comprehensive listing of physicians and hospitals is available on this website, as well as consultation numbers, basic physician information, and service ratings for each physician. All these kinds of information can be easily collected, because they are high-frequency data. Consequently, haodf.com is an appropriate website that can provide sufficient conditions for our research.

To establish a longitudinal timeline, this study developed a Python-based web crawler program, randomly selecting a six-month period (April 2022 to September 2022) to continuously collect data from the homepages of physicians nationwide who treat the specified diseases. The data encompasses information from physicians treating 14 common diseases in China. After cleaning the data and removing blanks, this study obtained information from 2,676 physicians’ personal homepages and consulted historical information generated during this period. All information displayed on the homepage is collected, and no information related to the privacy of physicians is collected. Based on mortality rates, diseases can be categorized into two groups, high-mortality diseases and low-mortality diseases.

In this study, patients’ consultation is considered the dependent variable, defined as the total number of online consultations by the physician. The independent variables include knowledge popularity, measured by the total views of the physician’s free knowledge articles, and knowledge attractiveness, reflected by the conversion rate of followers, which is the monthly new followers divided by the monthly article readers. The moderating variable, knowledge quantity, is indicated by the number of free knowledge articles published by the physician. Ultimately, based on earlier studies (58), this study incorporated various control elements to address confounding influences originating from both patient and physician dimensions. Patient-level controls include disease severity (D_Severity), categorized as low or high. It is hypothesized that patient needs for physician services are influenced by the severity of their condition. Physician-level controls comprise; gender (Gender), identified as male or female; physician’s medical title (Title); hospital classification (H_type), distinguishing public from private institutions; hospital rankings (H_level) are categorized into three levels; and hospital specialization (H_Special), differentiating between specialized and general hospitals. These aspects highlight and physician’s proficiency and are crucial in patients’ physician selection within OHCs.

### 3.2 Model estimation

Test our hypotheses regarding the three-way interaction between knowledge popularity, knowledge attractiveness, knowledge quantity, and patients’ consultations. Our model uses ordinary least squares (OLS) regression with time and city fixed effects. Moreover, the robustness checks are conducted using dependent variable substitution (ratio of consulting volume to visiting volume). To explore the interaction effects of knowledge popularity, knowledge attractiveness, and knowledge quantity on patients’ consultation, this study verified the following three equations:


(1) $$\begin{array}{c} \mathrm{logConsul}t_{\mathrm{it}}=\alpha_{0}+\beta_{1}\log{\left(\mathrm{Popular}\right)}_{\mathrm{it}}+\beta_{2}\log{\left(\mathrm{Attract}\right)}_{\mathrm{it}}\\ +\beta_{3}\mathrm{Titl}e_{\mathrm{it}}+\beta_{4}D\_\mathrm{severit}y_{\mathrm{it}}+\beta_{5}\mathrm{Gende}r_{\mathrm{it}}\\ +\beta_{6}H\_type_{\mathrm{it}}+\beta_{7}H\_\mathrm{leve}l_{\mathrm{it}}\\ +\beta_{9}H\_\mathrm{specia}l_{\mathrm{it}}+\mu_{i}+\lambda_{t}+E_{\mathrm{it}}\; \end{array}$$

(2) $$\begin{array}{c} \mathrm{logConsul}t_{\mathrm{it}}=\alpha_{0}+\beta_{1}\log{\left(\mathrm{Popular}\right)}_{\mathrm{it}}+\beta_{2}\log{\left(\mathrm{Attract}\right)}_{\mathrm{it}}\\ +\beta_{3}\log{\left(\mathrm{Popular}\right)}_{\mathrm{it}}*\log{\left(\mathrm{Attract}\right)}_{\mathrm{it}}+\beta_{4}\mathrm{Titl}e_{\mathrm{it}}\\ +\beta_{5}D\_\mathrm{severit}y_{\mathrm{it}}+\beta_{6}\mathrm{Gende}r_{\mathrm{it}}+\beta_{7}H\_type_{\mathrm{it}}\\ +\beta_{8}H\_\mathrm{leve}l_{\mathrm{it}}+\beta_{9}H\_\mathrm{specia}l_{\mathrm{it}}+\mu_{i}+\lambda_{t}+E_{\mathrm{it}}\; \end{array}$$

(3) $$\begin{array}{c} \mathrm{logConsul}t_{\mathrm{it}}=\alpha_{0}+\beta_{1}\log{\left(\mathrm{Popular}\right)}_{\mathrm{it}}+\beta_{2}\log{\left(\mathrm{Attract}\right)}_{\mathrm{it}}\\ +\beta_{3}\log{\left(\mathrm{Quantity}\right)}_{\mathrm{it}}+\beta_{4}\log{\left(\mathrm{Popular}\right)}_{\mathrm{it}}*\log{\left(\mathrm{Attract}\right)}_{\mathrm{it}}\\ +\beta_{5}\log{\left(\mathrm{Attract}\right)}_{\mathrm{it}}*\log{\left(\mathrm{Quantity}\right)}_{\mathrm{it}}\\ +\beta_{6}\log{\left(\mathrm{Popular}\right)}_{\mathrm{it}}*\log{\left(\mathrm{Quantity}\right)}_{\mathrm{it}}\\ +\beta_{7}\log{\left(\mathrm{Popular}\right)}_{\mathrm{it}}*\log{\left(\mathrm{Attract}\right)}_{\mathrm{it}}*\log{\left(\mathrm{Quantity}\right)}_{\mathrm{it}}\\ +\beta_{8}\mathrm{Titl}e_{\mathrm{it}}+\beta_{9}D\_\mathrm{severit}y_{\mathrm{it}}+\beta_{10}\mathrm{Gende}r_{\mathrm{it}}\\ +\beta_{11}H\_type_{\mathrm{it}}+\beta_{12}H\_\mathrm{leve}l_{\mathrm{it}}+\beta_{13}H\_\mathrm{specia}l_{\mathrm{it}}\\ +\mu_{i}+\lambda_{t}+E_{\mathrm{it}}\; \end{array}$$

City and time effects are denoted by $\mu_{i}$ and $\lambda_{t}$, respectively, with $\alpha_{0}$ representing the constant term and $E_{\mathrm{it}}$ indicating the residual error term. To address the issue of skewed distributions, log transformations were applied to the variables Consult (skewness = 9.095), Popular (skewness = 72.353), Attract (skewness = −55.620), and Quantity (skewness = 13.520). In Equation 1, the positive impact of *Popular* and *Attract* on *Consult* is assessed. Equation 2 investigates the moderating effect of *Attract* on the relationship between *Popular* and *Consult*. Equation 3 considers the three-way interaction among *Popular, Attract,* and *Quantity* on *Consult*. To mitigate potential multicollinearity issues among interaction terms, the variance inflation factor was computed.

## 4 Results

Table 1 presents descriptive statistics for the variables studied. Table 2 presents the Pearson correlation for the key variables used in this study. The findings reveal that the variance inflation factor statistics for all independent variables were below the threshold (VIF = 1.19), suggesting that multicollinearity is not a serious problem.

**Table 1:** Descriptive statistics.

	Variable descriptions	Mean	SD	Min	Max
Consult	Physician’s total number of online consultations	52.61	82.52	0.00	2698.00
Popular	Total views of physician’s free knowledge articles	3731.57	42467.97	−373000.00	4118000.00
Attract	Physician’s health account fan conversion rate, which is the monthly new followers divided by the monthly article readers.	8.94	120.83	−9504.00	1000.00
Quantity	Number of physician’s free knowledge articles	1.19	7.54	0.00	208.00
Title	Physician’s medical title classification is as follows: 4 represents Chief Physician, 3 corresponds to Associate Chief Physician, 2 indicates Attending Physician, 1 signifies Resident Physician.	3.27	0.74	1.00	4.00
D_severity	Disease severity (0 = low, 1 = high)	0.38	0.48	0.00	1.00
Gender	Physician’s gender (0 = male, 1 = female)	0.32	0.47	0.00	1.00
H_type	Physician’s Hospital Type (0 = Private Hospital, 1 = Public Hospital)	0.99	0.07	0.00	1.00
H_special	Physician’s hospital specialization level (0 = specialized hospital, 1 = general hospital)	0.68	0.47	0.00	1.00
H_level	Physician’s hospital grade (1 = primary level, 2 = secondary level, 3 = tertiary level)	2.99	0.10	2.00	3.00

**Table 2:** Correlation coefficient matrix.

	1	2	3	4	5	6	7	8	9	10
log(Consult)	1.000									
log(Popular)	0.578^***^	1.000								
	(0.000)									
log(Attract)	0.223^***^	−0.539^***^	1.000							
	(0.000)	(0.000)								
log(Quantity)	0.241^***^	0.325^***^	−0.131^***^	1.000						
	(0.000)	(0.000)	(0.000)							
Title	0.056^***^	0.030^***^	0.022^*^	0.015^*^	1.000					
	(0.000)	(0.001)	(0.010)	(0.085)						
D_severity	−0.025^**^	−0.000	−0.008^*^	0.016^*^	0.066^***^	1.000				
	(0.004)	(0.973)	(0.353)	(0.059)	(0.000)					
Gender	−0.036^***^	−0.109^***^	0.083^***^	−0.072^***^	0.081^***^	−0.147^***^	1.000			
	(0.000)	(0.000)	(0.000)	(0.000)	(0.000)	(0.000)				
H_type	0.001	−0.021^*^	0.011^*^	−0.047^***^	−0.013^*^	0.028^***^	−0.023^**^	1.000		
	(0.876)	(0.018)	(0.202)	(0.000)	(0.090)	(0.000)	(0.003)			
H_special	−0.005	−0.000	−0.003	0.003	0.023^**^	0.074^***^	−0.061^***^	0.077^***^	1.000	
	(0.568)	(0.991)	(0.770)	(0.696)	(0.004)	(0.000)	(0.000)	(0.000)		
H_level	0.028^**^	0.019^*^	0.003	0.017^*^	−0.007^*^	0.044^***^	−0.058^***^	0.149^***^	0.058^***^	1.000
	(0.001)	(0.030)	(0.702)	(0.055)	(0.394)	(0.000)	(0.000)	(0.000)	(0.000)	

*p-*values in parentheses. ^*^p < 0.05, ^**^p < 0.01, ^***^p < 0.001.

### 4.1 Hypotheses testing results

Table 3 presents the main ordinary least squares (OLS) regression models (models 1 to 4). Model 1 introduces control variables, showing that most of these variables are significant. Model 2, which includes the independent variables, indicates that log (*Popular*; *β* = 0.282, *p* < 0.001) and log (*Attract*; *β* = 0.547, *p* < 0.001) have significant and positive effects, supporting H1 and H2. When the moderating variable is added in model 3, the interaction between log (*Popular*) and log (*Attract*; *β* = 0.379, *p* < 0.001) is positive and significant, supporting H3. In contrast, the interactions between log (*Popular)* and log (*Quantity*; *β* = −0.040, *p* < 0.001) and between log (*Attract*) and log (*Quantity*; *β* = −0.084, *p* < 0.001) are negative and significant. Model 4 confirms the significant relationship among log (*Popular*), log (*Attract*), and log (*Quantity*; *β* = 0.182, *p* < 0.001), supporting H4.

**Table 3:** Regression result.

	Main models				Robustness models		
	Model 1	Model 2	Model 3	Model 4	Model 5	Model 6	Model 7
Constant	−1.772^***^	−1.039^***^	−1.015^***^	−0.995^***^	−0.245^*^	−0.225^*^	−0.203^*^
	(0.26)	(0.23)	(0.22)	(0.22)	(0.28)	(0.27)	(0.27)
Title	0.152^***^	0.071^***^	0.081^***^	0.081^***^	0.053^***^	0.063^***^	0.063^***^
	(0.01)	(0.01)	(0.01)	(0.01)	(0.01)	(0.01)	(0.01)
D_severity	−0.141^***^	−0.090^***^	−0.083^***^	−0.082^***^	−0.091^***^	−0.083^***^	−0.082^***^
	(0.02)	(0.01)	(0.01)	(0.01)	(0.02)	(0.02)	(0.02)
Gender	−0.166^***^	−0.046^**^	−0.070^***^	−0.071^***^	−0.075^***^	−0.101^***^	−0.101^***^
	(0.02)	(0.02)	(0.01)	(0.01)	(0.02)	(0.02)	(0.02)
H_type	−0.028	0.178^*^	0.127^*^	0.123^*^	0.252^*^	0.194^*^	0.190^*^
	(0.11)	(0.10)	(0.09)	(0.09)	(0.12)	(0.11)	(0.11)
H_special	−0.017^*^	−0.021^*^	−0.023^*^	−0.024^*^	−0.013	−0.015^*^	−0.016^*^
	(0.02)	(0.02)	(0.01)	(0.01)	(0.02)	(0.02)	(0.02)
H_level	0.449^***^	0.234^**^	0.241^***^	0.236^***^	−0.004	0.007	0.001
	(0.08)	(0.07)	(0.07)	(0.07)	(0.09)	(0.09)	(0.09)
log(Popular)		0.282^***^	0.327^***^	0.326^***^	0.261^***^	0.308^***^	0.307^***^
		(0.00)	(0.00)	(0.00)	(0.01)	(0.01)	(0.01)
log(Attract)		0.547^***^	0.664^***^	0.662^***^	0.517^***^	0.636^***^	0.634^***^
		(0.01)	(0.01)	(0.01)	(0.01)	(0.01)	(0.01)
log(Quantity)			0.411^***^	0.245^***^		0.434^***^	0.256^**^
			(0.06)	(0.06)		(0.07)	(0.08)
log(Popular)*log(Attract)			0.379^***^	0.368^***^		0.394^***^	0.383^***^
			(0.01)	(0.01)		(0.01)	(0.01)
log(Popular)*log(Quantity)			−0.040^***^	−0.026^***^		−0.044^***^	−0.028^***^
			(0.01)	(0.01)		(0.01)	(0.01)
log(Attract)*log(Quantity)			−0.084^***^	−0.053^**^		−0.056^*^	−0.023^*^
			(0.02)	(0.02)		(0.02)	(0.02)
log(Popular)*log(Attract)*log(Quantity)				0.182^***^			0.195^***^
				(0.03)			(0.03)
City dummies	Yes	Yes	Yes	Yes	Yes	Yes	Yes
Time dummies	Yes	Yes	Yes	Yes	Yes	Yes	Yes
*R* ^2^	0.727	0.793	0.818	0.819	0.638	0.670	0.671
adj. *R*^2^	0.727	0.792	0.818	0.819	0.637	0.670	0.671
*N*	13,380	13,380	13,380	13,380	13,380	13,380	13,380

Standard errors in parentheses. ^*^p < 0.05, ^**^p < 0.01, ^***^p < 0.001.

### 4.2 Robustness check

Since consultations follow visits, the total number of visits serves as a viable alternative in models 5, 6, and 7 for robustness checks. Model 5 shows that log (*Popular*; *β* = 0.261, *p* < 0.001) and log (*Attract*; *β* = 0.517, *p* < 0.001) are positively and significantly related to patients’ consultations. Model 6 finds the interaction term of log (*Popular*) and log (*Attract*; *β* = 0.394, *p* < 0.001) to be positive and significant, while the interaction terms between log (*Popular*) and log (*Quantity*; *β* = −0.044, *p* < 0.001) and between log (*Attract*) and log (*Quantity*; *β* = −0.056, *p* < 0.05) are negative and significant. Model 7 indicates that the interaction term among log (*Popular*), log (*Attract*), and log (*Quantity*; *β* = 0.195, *p* < 0.001) is significant. Thus, the robustness checks in Table 3 corroborate the main models.

**Figure 2:**
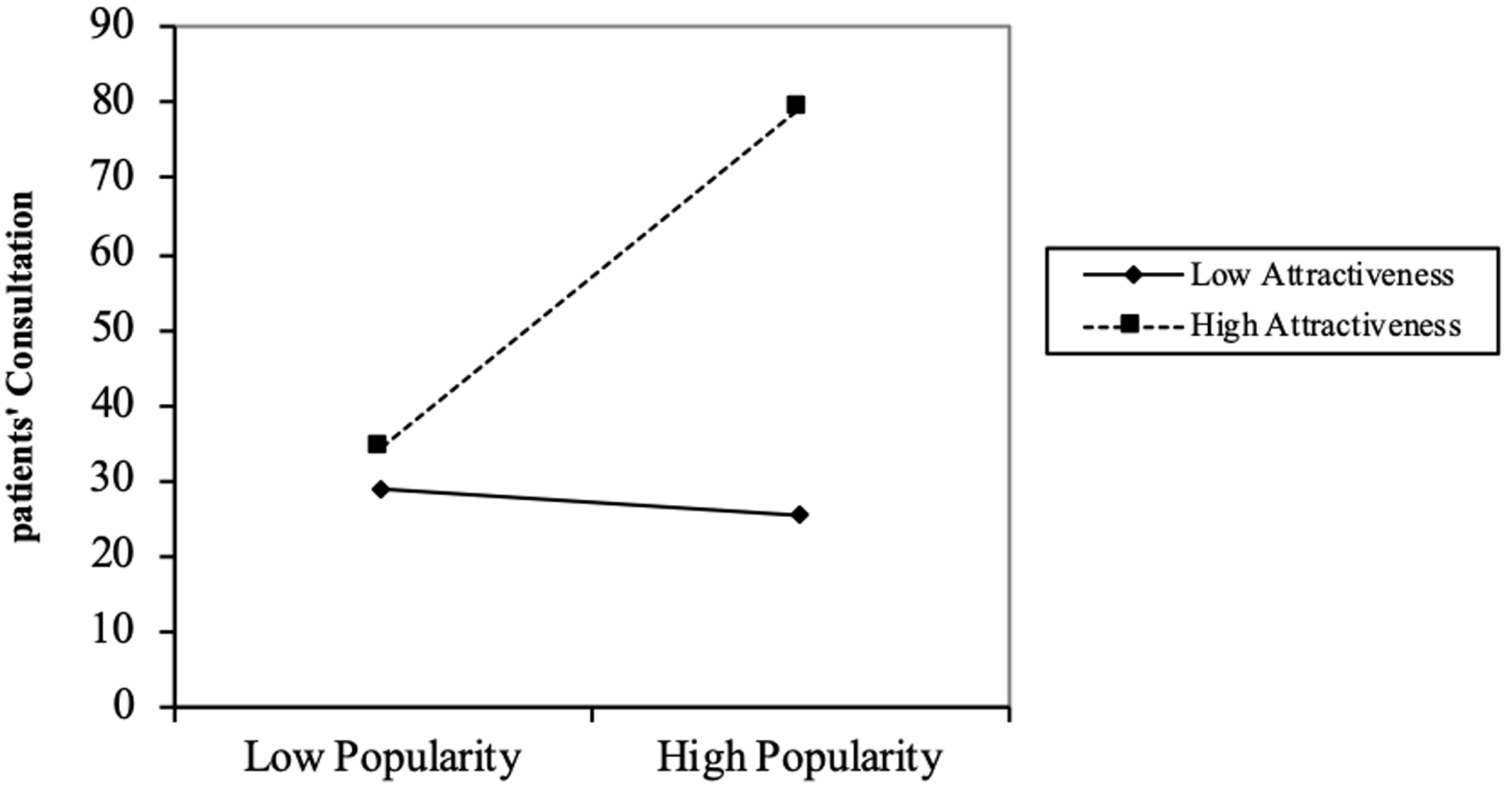
Interaction of popularity and attractiveness on patients.

Figure 2 illustrates that for physicians with high knowledge attractiveness, increased knowledge popularity leads to more patient consultations. When knowledge attractiveness is high, the increase in the independent variable (knowledge popularity) results in a more noticeable growth in the dependent variable (patients’ consultation). The results indicate a strong positive moderating effect of knowledge attractiveness, confirming Hypothesis 3.

**Figure 3:**
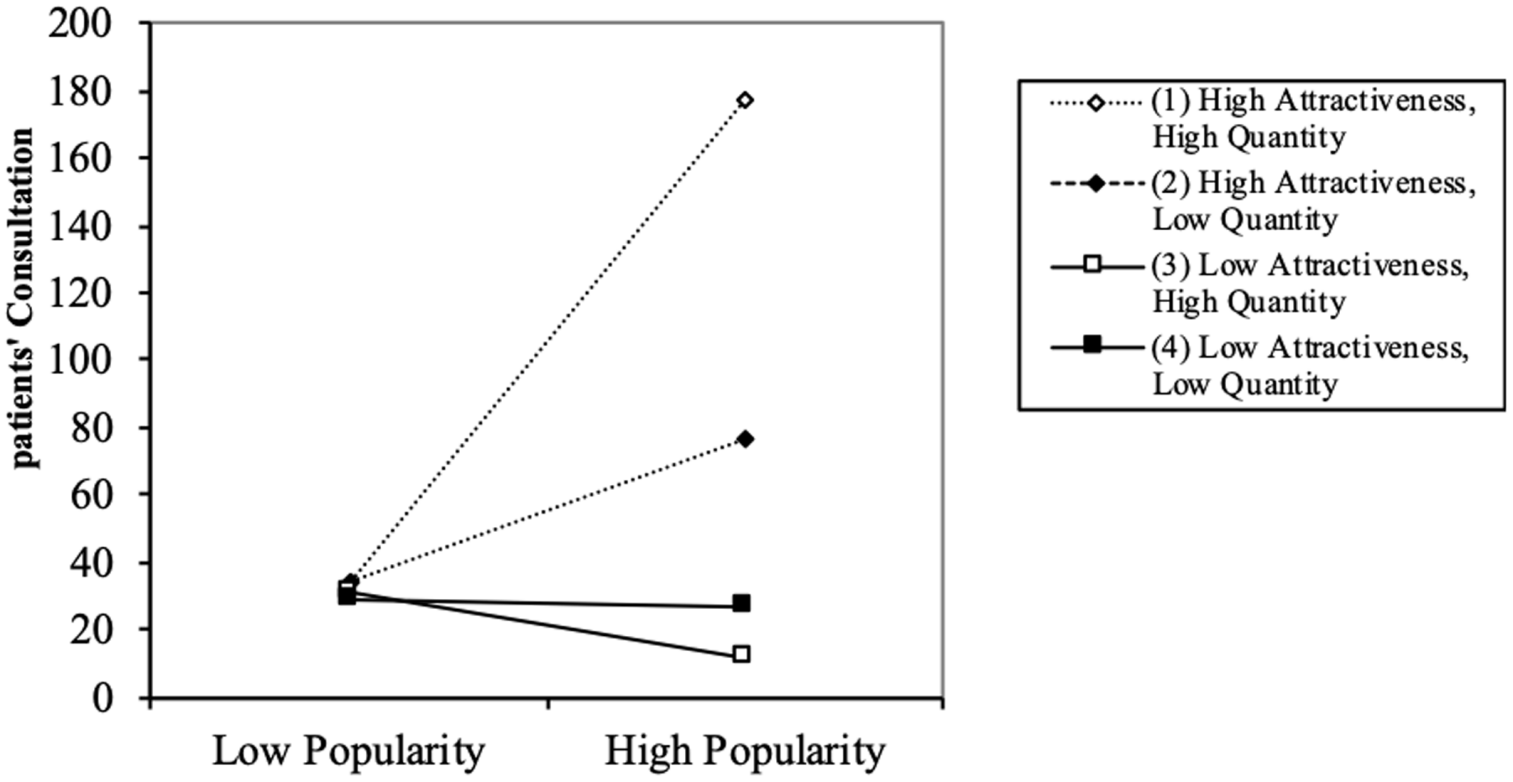
Three-way interaction of popularity, attractiveness and quantity on patients’ consultation.

The three-way interaction effect is shown in Figure 3. The positive moderating effect of knowledge attractiveness is expected to be strengthened at high rather than low knowledge quantity. Figure 3 demonstrates that the positive effect of knowledge popularity on patients’ consultation is significantly amplified when both knowledge attractiveness and knowledge quantity are high, thereby validating Hypothesis 4.

## 5 Discussion

This study aims to explore the direct and interactive effects of peer recognition cues from other patients, specifically knowledge popularity and knowledge attractiveness, on patient consultation behavior. Additionally, it investigates the three-way interaction of peripheral cues—physician’s knowledge quantity, knowledge popularity, and knowledge attractiveness—on patient consultation behavior. These findings provide significant insights for community engagement and sustainable development in OHCs.

The research results showed that both knowledge popularity and knowledge attractiveness have a significantly positive impact on patient consultation behavior. The confirmation of Hypotheses 1 and 2 was obtained. This result is consistent with previous research literature, which have suggested that knowledge popularity and knowledge attractiveness have positive effects on consumer decision-making and community engagement. For example, the previous study suggested that knowledge popularity influences consumer decision-making, particularly for unfamiliar products (59). Because popular products are viewed more favorably, this plays a role in affecting consumer choices.

In addition, this study’s results show trusted support in the theory that knowledge attractiveness significantly moderates the relationship between knowledge popularity and patient consultation behavior. Knowledge attractiveness enhances the positive relationship between knowledge popularity and patient consultation behavior. The confirmation of Hypotheses 3 was obtained. Prior research has investigated the significant positive impact of post popularity and attractiveness on user participation behaviors in social media marketing activities (such as liking and sharing information) (60). The results of this study are consistent with previous research in social media contexts, but further explore the interaction effects of these two factors on user participation behavior in OHCs. Furthermore, the study found that the positive moderating effect of knowledge attractiveness is contingent upon the level of knowledge quantity of the physician. The positive moderating effect of knowledge attractiveness on patient consultation behavior is amplified when the knowledge quantity is high. This suggests that the higher the number of articles shared by the physician, the more likely that patients perceive the need to seek peer recognition from other patients. The confirmation of Hypotheses 4 was obtained. Previous studies have confirmed the significant positive impact of knowledge quantity on user community loyalty and satisfaction (61). The results of this study not only validate the positive effects of knowledge quantity but also delve deeper into its complex interaction effects with other cues.

## 6 Theoretical implication

The study provides theoretical innovation in the realm of OHCs, patient engagement and digital communication. This research deepens our understanding of the deeply layered processes that unfold in patient consultation and information exchange in the digital healthcare space (17).

First, this study test the direct and interactive effects of knowledge popularity and knowledge attractiveness (two forms of peer recognition) on patient consultation engagement in the OHC environment. This research extends the utility and application of peer recognition theory to an OHC setting. While previous research has often focused on the individual effects of knowledge popularity or knowledge attractiveness (45, 48), few studies have examined their interactive effects. This research makes a significant contribution to the understanding of peer recognition in OHCs.

Second, our findings indicate that peripheral cues significantly influence patient decisions. Specifically, knowledge popularity has the strongest positive impact on patient consultations when both knowledge attractiveness and the quantity of knowledge articles are high. These results provide new insights into the mechanisms of the peripheral route in ELM. This study aligns with previous literature which positions source attractiveness as a crucial peripheral cue (62). Additionally, the previous study also found that social cues significantly impact online health information seeking behaviors. By integrating these insights, our research adds depth to the understanding of how peripheral cues affect patient decision-making in online health communities (63).

## 7 Practical implication

This study makes valuable practical sharing to the realm of digital healthcare by providing insights into the dynamics of patient-physician interactions within online healthcare communities (OHCs). The findings of this research carry implications for healthcare professionals, platform designers, and policymakers, aiming to optimize patient engagement and knowledge sharing in the digital era.

First, this study offers implications for platforms that are pursuing sustainability. Platforms can use the findings of this study to understand the mechanisms that can stimulate active knowledge contribution by physicians on OHCs. If platforms recognize the role of peer recognition, popularity and attractiveness of knowledge, the amount of knowledge, and interactions between these factors, they can tailor knowledge creation strategies to drive engagement and maintain physician retention on their platform. In doing so, platforms can help physicians attract more consultations, participants, and patient consultations, and ultimately grow an engaged and sustainable OHC.

Second, medical education institutions can incorporate the findings into their curricula, training the next generation of physicians for effectual knowledge sharing in the digital age. By understanding the subtlety of peer recognition and its impact on patient engagement, medical professionals can be better prepared in their interactions with patients online and offline.

## 8 Limitations and future research

This study also has limitations. First, the data used in this study is based on a single online health care platform, haodf.com. Future studies could benefit from expanding the sample to include multiple platforms to enhance the generalizability of the findings. Second, the scope of diseases considered in this study is limited. Due to resource constraints, data were collected from physicians specializing in only 14 types of diseases. Future research could explore a broader range of diseases to determine if patient consultation behaviors vary across different medical conditions. Lastly, the limitations of web scraping mean that some relevant information might have been inaccessible, potentially affecting the study’s outcomes. Future research should aim to supplement these missing data points by incorporating additional factors that could influence patient consultation behaviors.

## 9 Conclusion

This study utilizes the Elaboration Likelihood Model (ELM) and Information Overload Theory to examine the three-way interaction effects of peripheral cues—knowledge popularity, knowledge attractiveness, and knowledge quantity—on patient consultation behavior within the context of OHCs. The results indicate that peer recognition cues (knowledge popularity and knowledge attractiveness) from other patients have positive impacts on patient consultation behavior. Additionally, the study finds that knowledge attractiveness positively moderates the relationship between knowledge popularity and patient consultation behavior. Furthermore, the study confirms that the three-way interaction significantly affects patient consultation behavior, with the positive moderating effect of knowledge attractiveness being strengthened when the quantity of shared knowledge is high. Using 6 months of online scraping data from the haodf.com, the study validates the three-way interaction model. These insights provide valuable guidance for platform design and healthcare practitioners, enhancing patient engagement and establishing a sustainable knowledge-sharing ecosystem on digital health platforms.

## Data Availability

The raw data supporting the conclusions of this article will be made available by the authors, without undue reservation.

## Footnotes

^1^ www.haodf.com/
